# Genetics and genomics in Brazil: a promising future

**DOI:** 10.1002/mgg3.95

**Published:** 2014-07-01

**Authors:** Maria Rita Passos-Bueno, Debora Bertola, Dafne Dain Gandelman Horovitz, Victor Evangelista de Faria Ferraz, Luciano Abreu Brito

**Affiliations:** 1 Centro de Pesquisa sobre o Genoma Humano, Departamento de Genética e Biologia Evolutiva, Instituto de Biociências Universidade de São PauloSão Paulo, Brazil; 2 Instituto da Criança do Hospital das Clínicas da Faculdade de Medicina Universidade de São PauloSão Paulo, Brazil; 3 Centro de Genética Médica, Instituto Nacional de Saúde da Mulher, daCriança e do Adolescente Fernandes Figueira, Fundação Oswaldo CruzRio de Janeiro, Brazil; 4 Departamento de Genética, Faculdade de Medicina de Ribeirão Preto, Universidade de São PauloRibeirão Preto, São Paulo, Brazil


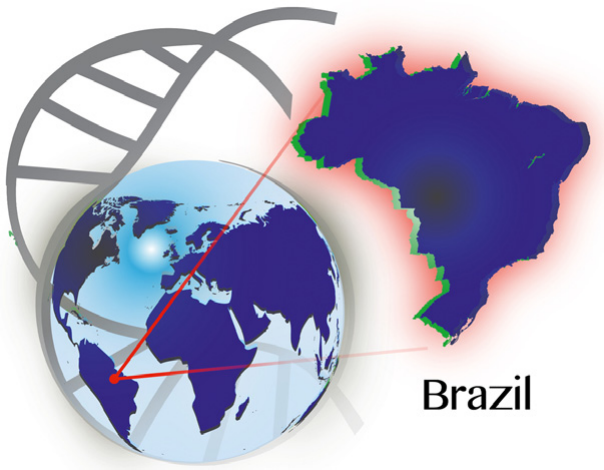


## Introduction

Medical Genetics has a recent history in Brazil and started out as a research program in Human Genetics during the 1950s, mainly by a shift in work of Brazilian scientists from genetic studies conducted on *drosophila melanogaster* to this new emerging field of research, following a worldwide tendency (Otto and Frota-Pessoa 1975; Beiguelman 2000). The Department of Biology, currently Department of Genetics and Evolutionary Biology, at the Institute of Biosciences, University of São Paulo, was one of the main pioneers in this field, followed by other initiatives at the Federal Universities at Rio Grande do Sul and Paraná (Otto and Frota-Pessoa 1975; Beiguelman 2000). The importance of genetics was recognized by the medical community only as of the 60 and onward, after the advent of human cytogenetics, and with the introduction of Genetics as a subject in a few medical schools (Beiguelman 2000). Nevertheless, Medical Genetics still is regarded as an optional discipline in many medical schools in Brazil.

The Brazilian population is highly heterogeneous and admixed, as a result of five centuries of crossbreeding among native Amerindians, Europeans settlers and immigrants, and sub-Saharan Africans, who were mostly brought to this country during the slavery period. The country's diverse regions underwent slightly different colonization processes: the North region had a larger preservation of indigenous people, the Northeast region had a stronger history of African influence, while Europeans colonized most of the South region (Alves-Silva et al. 2000; Pena et al. 2011). More recently, waves of Asian immigration reached mainly the Southeastern region. This trihybrid admixture process was strongly influenced by a directional mating between European males and African and Amerindian females during the colonization period, as demonstrated by the use of sexual and mitochondrial chromosome markers (Pena et al. 2009; Resque et al. 2010). In the last decade, the availability of genomic ancestry informative markers (AIMs) has encouraged a precise characterization of the contribution of each parental population. It has been shown that European ancestry is evenly preponderant across the country; the African contribution has reached the highest proportion in the Northeast (∼30.3%), followed in decreasing order by the Southeast (∼18.9%), South (∼12.7%), and North (∼10.9%) regions, while the Amerindian contribution is the highest in the North (∼19.4%) region, and relatively evenly spread across the other regions (Santos et al. 2010; Pena et al. 2011). An unexpected high Amerindian contribution is also found in semi-isolated communities founded by African-slaves refugees, the “quilombos” (Lopes Maciel et al. 2011; Kimura et al. 2013; Gontijo et al. 2014). Our colonization history also accounts for the high incidence of some diseases. For example, the autosomal recessive (AR) sickle cell disease (SCD), one of the most common monogenic disorder in Brazil (Cançado and Jesus 2007; Lobo et al. 2014, Table 1), has been attributed to the intense African slave trade that occurred between the 16th and 19th centuries (Aygun and Odame 2012).

**Table 1:** Genetic disorders under federal clinical protocols and guidelines for treatment including those from the newborn screening

Disease	Treatment	Ministry of Health official programs updated	Overall estimated prevalence^3^
Included in newborn screening			
Congenital adrenal hyperplasia	Replacement hormone medication	2010	1:7500–1:17,091^4^ (Brazil)
Congenital Hypothyrodism^1^	Levothyroxine^2^	2010	1:3808 (Brazil)
Cystic fibrosis	Pancreatic enzymes; dornase alfa	2010	1:7000^5^–1: 13,073 (Brazil)
Sickle cell anemia	Hydroxyurea	2010	1:1000^6^–1:3129 (Brazil)
Phenylketonuria	Dietary supplement	2013	1:24,780 (Brazil)
Biotinidase deficiency	Biotin^2^	–	1:61,067^7^
Not included in newborn screening			
Gaucher	Enzyme replacement	2011	1:57,000^8^
Hereditary angioedema	Danazol	2010	1:10,000–1:50,000^9^
Hereditary ichthyosis	Retinoid	2010	1:4000 males (X linked form – Germany)^10^
Osteogenesis Imperfecta	Bisphophonates	2013	0.74:10,000^11^
Primary immunodeficiencies: antibody deficiencies	Immunoglobulin	2007	0.12:100,000^12^
Turner syndrome	Growth hormone	2010	1:1500–1:2500 females^13^
Wilson disease	Chelation therapy, zinc acetate	2013	1:30,000^14^

1 Included in the newborn screening despite not being a genetic disorder.

2 The medication is available in SUS, although there is no clinical protocol established.

3 Brazilian Society of Newborn Screening (SBTN). In general, it was used data from the SBTN; prevalence estimates, however, varies across different data sets.

4 Silveira et al. (2008), Pezzuti et al. (2014).

5 Raskin et al. (2008).

6 Cançado and Jesus (2007).

7 Wolf (1991).

8 Meikle et al. (1999).

9 Bork et al. (2003).

10 Traupe et al. (2014).

11 Prevalence based on South America countries (Barbosa-Buck et al. (2012)).

12 Leiva et al. (2007).

13 Saenger (1996).

14 Bachmann et al. (1991).

The Brazilian admixture, as briefly explained, constitutes a good example of a population characteristic boosting the research on Genetics in Brazil. Inbreeding in Brazil played a similar role within this field. The cultural diversity, socio-economic status, migration, population density, urbanization, and permissive laws have historically influenced the heterogeneous degree of consanguineous marriages across the country (Machado et al. 2013; Freire-Maia 1957, Fig. 1A). Even though inbreeding has been decreasing in all Brazilian regions in the past decades, large differences between Southern and Northeastern populations still remain, with the coefficient of inbreeding (*f*) 13 times higher in some Northeastern (*f* = 0.00395; proportion of consanguineous marriage from 6% to 12%) populations as compared to Southern (*f* = 0.0003) populations (Freire-Maia 1957, 1989; Brito et al. 2011; Weller et al. 2012; Machado et al. 2013), where the frequency of consanguineous marriage (<5%) is comparable to the estimates found for most of the other countries around the world (Hamamy et al. 2011).

**Figure 1:**
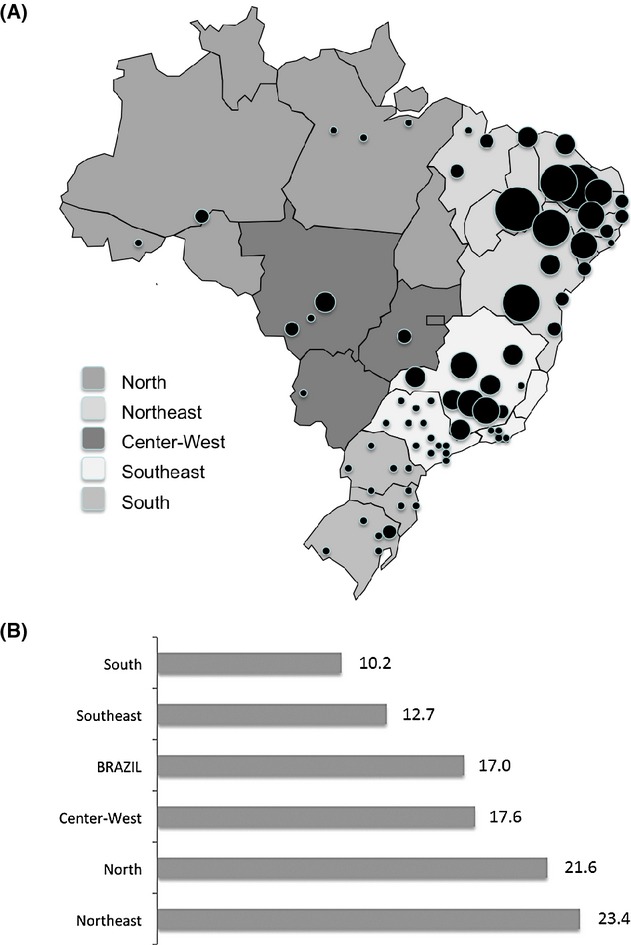
Inbreeding and Infant mortality rate across Brazil; (A) Inbreeding occurrence in Brazil, adapted from Salzano and Freire-Maia 1967. The diameter of black dots are proportional to the coefficient of inbreeding (*f*). According to that study, Brazil has a high-inbreeding zone, with *f* values between 0.007–0.01, spanning most of Northeastern inland, a medium-inbreeding zone (*f* values between 0.002–0.005), in the Northeastern coast and some Central–Western areas, and a low-inbreeding zone (*f* values below 0.001), encompassing the Southern states and other sparse areas.; (B) Infant mortality rate in Brazilian regions (per 1000), according to the 2010 Census data of Brazilian Institute of Geography and Statistics (IBGE).

It is widely accepted that consanguineous marriage is a risk factor for AR and multifactorial inheritance disorders. Indeed, Brazilian consanguineous families have been useful study subjects for identifying pathogenic mutations underlying AR disorders, some of them originally described in Brazil, such as acheiropodia, Richieri-Costa-Pereira syndrome and Spastic paraplegia, optic atrophy, and neuropathy (SPOAN, Freire-Maia et al. 1975; Ianakiev et al. 2001; Macedo-Souza et al. 2005; Guion-Almeida et al. 2009; Lines et al. 2012; Favaro et al. 2014), in addition to others already described, such as Knobloch syndrome (Sertié et al. 2000). Still, high rates of inbreeding were recently reported to increase prevalence of some rare AR disorders, such as mucopolysaccharidoses and short stature, among others, in small Brazilian villages (Salvatori et al. 1999; Costa-Motta et al. 2011). Recently, National Institute of Populational Genetics (INAGEMP) has launched a census concerning clusters of genetic disorders in Brazil. Preliminary data list more than 100 small villages with a likely higher prevalence of a specific disorder. At a national level, however, the degree of inbreeding in Brazil has never been associated with increased overall prevalence of AR disorder. The impact of inbreeding rate in the etiology of complex disorders in the Brazilian population is still quite unexplored. Nevertheless, it has been shown to confer increased risk for hypertension in quilombos (Kimura et al. 2012), but not for nonsyndromic cleft lip and palate (Brito et al. 2011).

The population admixture has always posed a challenge to the gene mapping studies of complex traits in Brazil, including pharmacogenomic studies, particularly when designing case–control studies, which are prone to produce spurious association in the presence of population structure. Accordingly, AIMs have proven to be useful for inferring the putative biogeographic ancestry of individuals, estimating the ancestry proportions of admixed individuals and populations (Shriver et al. 1997; Halder et al. 2008; Pena et al. 2011; Pereira et al. 2012; Suarez-Kurtz et al. 2012; Manta et al. 2013), correcting case–control studies for stratification bias (Brito et al. 2012a,b; Bagordakis et al. 2013), and performing admixture mapping approaches. Many of such studies have benefited from AIM panels built by Brazilian research groups, focused specifically on discriminating Brazilian parental populations (Bastos-Rodrigues et al. 2006; Santos et al. 2010; Brito et al. 2012a; Manta et al. 2013). In addition, in an ethnically admixed population, it is possible to evaluate the impact of certain at-risk alleles on the occurrence of some disorders or to drug response among different ethnicities simultaneously, under similar environmental and socio-economical conditions (Suarez-Kurtz et al. 2014).

**Figure 2:**
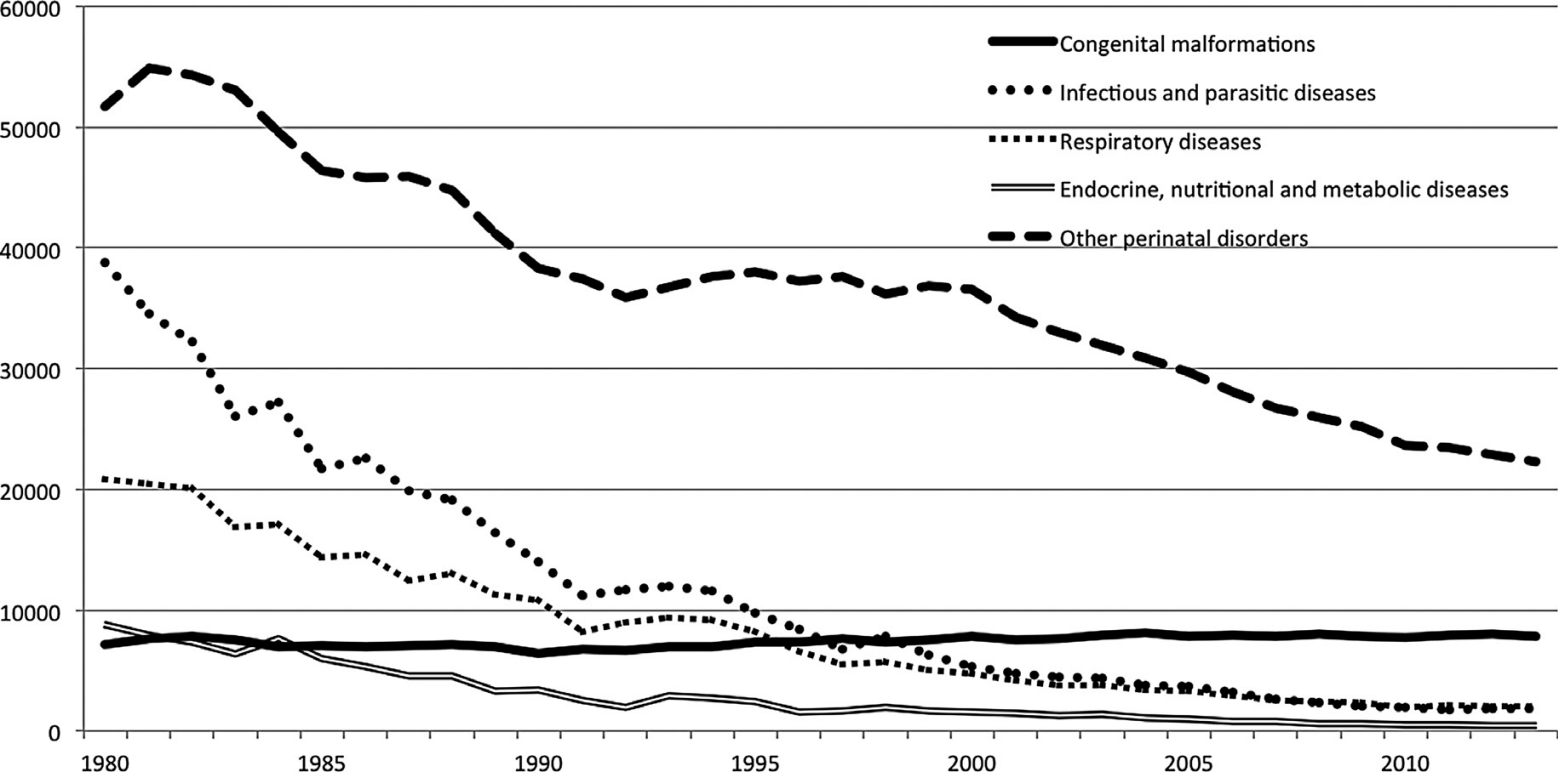
Evolution of infant mortality (under 1 year old) by year, from 1980 to 2013. With the observed overall decrease in deaths caused by different conditions, congenital malformations became the second cause of infant mortality in Brazil (DATASUS).

Brazil, with 26 states (Fig. 1A), is the world's 5th largest country in area (8,515,767 km^2^) and population (199,242,462 million individuals; IBGE, 2012). The age profile still remains as that of a young country, with 25% of the population under 15 years old and just 7% over 65 years old. Life expectancy at birth is estimated as 73.8 years old. Despite presenting the 7th largest gross domestic product (GDP), it ranks 61st and 85th in GDP per capita and in human development index (0.73), respectively (IBGE, 2012; reviewed in Acosta et al. 2013; World Bank, 2014, data.worldbank.org), reflecting an outstanding social inequality. As a related example, Brazil still has a high infant mortality rate, but it varies considerably across the country (Fig. 1B). On behalf of policies with impact on sanitation and health, this rate has progressively decreased, and as part of the Millenium Development Goals from the United Nations (UN), Brazil reached the goal of decreasing the mortality rate to 2/3 of the one observed in 1990, 2 years before the deadline, set for 2015 (United Nation Development Programme, 2014). In this scenario, congenital malformations have become the second most common cause of death in children under 1 year of age surpassing infectious diseases, and represents more than 15% of the total number of infant deaths since 2000 (Fig. 2; DATASUS, tabnet.datasus.gov.br). This data highlight the increasing importance of Genetics for the health of the Brazilian population.

In this manuscript, we focus on describing the genetic services provided in the country, how they are working and some of our laws with direct impact on the quality of life of families with rare genetic disorders, as well as including Federal government support for treatment of some rare genetic disorders.

## Health Care

The public unified health system (“Sistema Único de Saúde” or SUS) serves the majority of the Brazilian population. SUS was created by the Brazilian Federal Constitution in 1988 and it is organized by the Ministry of Health, with subsystems in each Brazilian State and city. SUS possibly represents the largest public health system in the world, and includes most aspects of health care, from outpatient care to organ transplantation, and proposes guidelines to ensure full, universal and free-of- charge medical access for the entire Brazilian population. In addition to offering appointments, medical exams and hospitalizations, the SUS also promotes immunization campaigns, prevention and health monitoring, such as food control and medicament registration (Brazilian Ministry of Health, 2014). It covers few genetic services, most particularly tests for genetic newborn screening, as detailed below.

In addition to SUS, 25.9% of the Brazilian population (49.2 million inhabitants) has some sort of private health insurance coverage (private health care plans). This percentage is much higher in urban (29.7%) than in rural areas (6.4%), and also higher in the Southeast/South regions (35.6/30.0%) than in the North/Northeast regions (13.3/13.2%) (IBGE, 2009). These companies are regulated by a National Supplementary Health Agency (ANS), a federal government organization established in 2000. Guidelines for coverage of several laboratory procedures for genetic diagnosis (genomic cytogenetics, biochemical and molecular techniques, particularly sequencing, and prenatal diagnostic tests) by the Insurance Companies were issued in January 2014.

## Genetic Services Provided

Genetic services started growing at research settings and universities. Despite the progressive translation of genetic research into a clinical field and efforts from the medical community toward its recognition as a specialty in SUS, only in 2014 did the Ministry of Healthy issue guidelines for complete assistance to be given to individuals with rare disorders (Policy of Rare Diseases, Box 1). Within this context, in the multidisciplinary team, medical Geneticists were contemplated as an integral part of it. Hopefully, with these national actions, the scenario presented here will improve within the next decade.

**Box 1**

The National Policy for Rare Diseases* in Brazil, defines guidelines for offering treatment to individuals affected by rare diseases in the public unified health system (Sistema Único de Saúde, SUS). It arose as a consequence of a great effort and pressure from citizens, aiming at reducing mortality and morbidity for individuals with rare diseases. The project defines an annual plan of action and financial and logistical support, and envisages the establishment of a national database (important for facilitating the access to high cost drugs, genetic tests, for instance) and the creation of reference treatment centers. These centers should be able to evaluate patients, perform genetic testing procedures, diagnose, treat and offer genetic counseling (Diario Oficial da União – ISSN 1677-7042no. 30, 12 February 2014, section 1, pages 44–55).

*The policy adopted the same definition of World Health Organization for rare diseases affecting 65 out of 100,000 individuals.

### Clinical services

Most public centers and care services related to the field of clinical genetics are usually located in State capitals, and concentrated in the Southeast and South of Brazil. They are usually integrated with public universities, teaching hospitals – some of them with excellence in specific areas such as Inborn Errors of Metabolism (IEM) within the Hospital das Clínicas de Porto Alegre (HCPOA), at Universidade Federal do Rio Grande do Sul - or hospitals specialized in specific disorders, such as the Hospital for Rehabilitation of Craniofacial Anomalies – Bauru, Universidade de São Paulo. These services are responsible for the medical care of thousands of individuals and families annually and in the case of HCPA, as well as for providing biochemical and enzymatic assays for the diagnosis of IEM for any patient in Brazil. These genetic services are usually regarded as reference centers at the regional or national level. There is, however, great difficulty in accessing them as there are no official databases.

**Figure 3:**
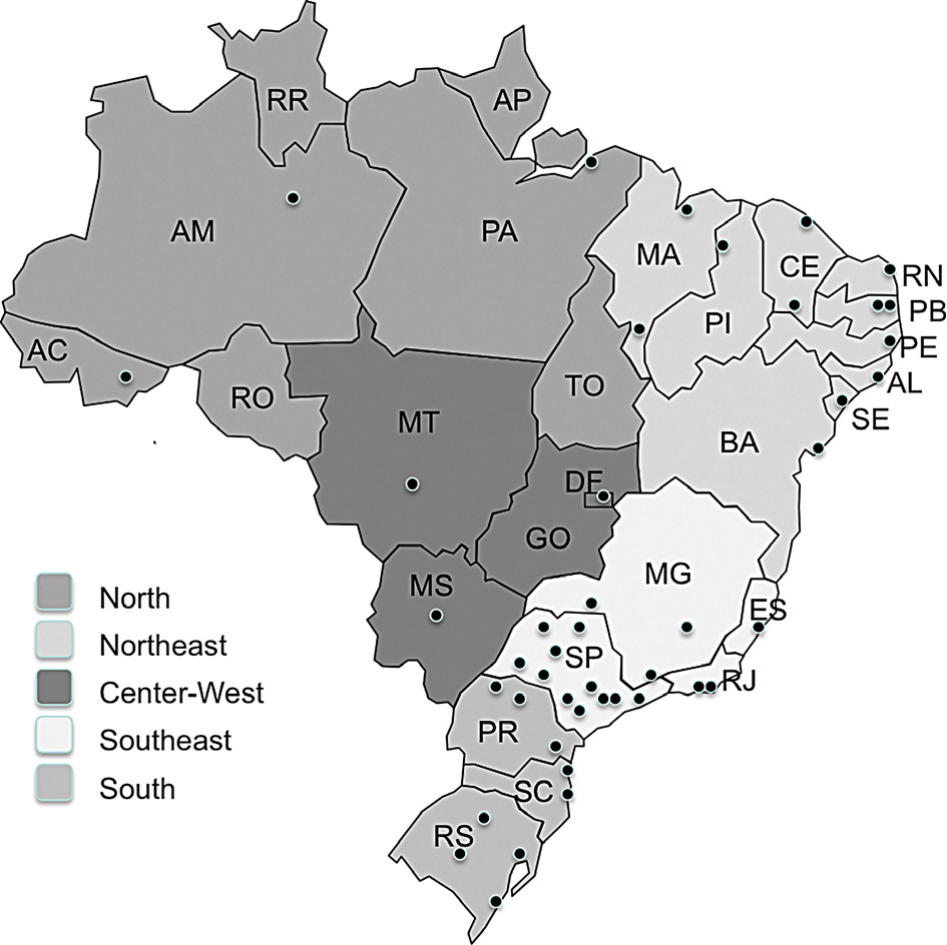
Updated graphic representation of clinical genetic services in Brazil (2012), with aggregated information from the census by Horovitz (2003), outpatient information system from the Ministry of Health and Brazilian Society of Medical Genetics, plus updated information on new clinical genetic services (presentation of abstracts at meetings of the Brazilian Society of Medical Genetics and personal communications between the years 2003 and 2009). There is no reference to any type of care in the speciality in the states of Amapá (AP), Roraima (RR), Rondônia (RO) and Tocantins (TO).

According to a census conducted in 2003 for mapping public medical genetic services, medical genetic clinical care was offered at 48 institutions in Brazil, and 33 of them were somehow integrated with a genetic laboratory (Horovitz 2003). More recent data show that new genetic services are being implemented (96 clinical genetic services: 66 in the Southeast/South regions and 21 in Northeast states), particularly in states previously devoid of such kind of assistance. Nevertheless, some Brazilian States (Amapá, Roraima, Rondônia and Tocantins, all of them located in the North region), still lacked specialized care in Genetics (Fig. 3).

Despite the increase in medical genetic care, the current number of public services offered and specialized personnel in medical and human genetics are far below the needs of the country (Horovitz et al. 2013; Acosta et al. 2013).

### Genetic laboratories

The availability of publically funded diagnostic exams for genetic diseases follows a distribution pattern similar to that of the public medical genetic services in Brazil, with a higher concentration in the South-Southeast regions (Horovitz 2003; Marques-de-Faria et al. 2004). Today, there is no logical referenced flow for public diagnostic tests and public clinical services around the country, instead informal agreements have been made between them.

A research study conducted on public genetic services in Brazil (Horovitz 2003, 2003) showed that diagnostic genetic tests were available in 47 of 66 genetic services, which are partly funded by SUS. Among them, 83% offered conventional cytogenetics, 55% high-resolution cytogenetics, 32% fluorescent in situ hybridization, 36% tests for IEM, and 32% prenatal diagnosis. About 50% of them also used molecular biology techniques for several groups of diseases, including mental retardation, dysmorphic syndromes, cancer predisposition, infertility, and neuromuscular and metabolic diseases, among others. The use of molecular biology techniques has grown and some groups have implemented the use of next generation sequencing (NGS) technology, but most of them were introduced by research and funded projects. Their permanence and use for diagnosis, however, has been challenged in many institutions as a result of lack of funding. However, this picture will change after the implementation of the policy for rare diseases, as specialized services will be encouraged to provide full care, including laboratory diagnosis, with the provision of specific Federal funding (Brazilian Ministry of Health, 2014).

In the private setting, genetic tests are more readily available, with the promptness of the investigation and access to complementary exams the main difference between private care and consultations in public hospitals. All kinds of testing procedures are available in the private sector, including not only the diagnosis of genetic disorders of affected individuals, but also pre-implantation genetic diagnosis, prenatal diagnosis, non-invasive prenatal diagnosis (NIPT), expanded newborn screening, predictive testing, and pharmacogenetics. The private sector usually makes use of the most advanced techniques, including the recent availability of exome and genome sequencing which became available in a few private laboratories in some of the State Capitals of Brazil. Nonetheless, despite the availability of these tests, samples are sometimes sent abroad for processing and health insurance companies do not always cover the costs.

## Professionals in Genetics

### Medical genetics residency

The medical practice in genetics in the country, as mentioned earlier, has had a recent onset, particularly when compared to other medical specializations. Although the first service that offered residency in Medical Genetics was established in 1977 in the Hospital of the Medical School of Ribeirão Preto, University of São Paulo, Ribeirão Preto, SP, only in 1983 did the Federal Council of Medicine regulate this Medical specialization. The Brazilian Society of Clinical Genetics (Sociedade Brasileira de Genética Clínica – SBGC), later renamed as Brazilian Society of Medical Genetics (Sociedade Brasileira de Genética Médica – SBGM), was established in 1986 at the 32nd National Genetics Meeting and the 38th Annual Meeting of the Brazilian Society for the Progress in Science (Sociedade Brasileira para o Progresso da Ciência) (Brunoni 1997). The program in Medical Genetics, approved by the National Medical Residence Committee, is a 3-year training residency program. Eligible candidates are individuals who have received a medical degree. The program includes training in specific areas of Internal Medicine and Pediatrics, in addition to Medical Genetics. State or Federal funds support the majority of residency training in Brazil. At the end of the program, the resident receives a specialization title issued by the Ministry of Education. In turn, the Brazilian Medical Genetics Society (SBMG) offers board certification in Medical Genetics. It is recognized by the Brazilian Medical Association and the Federal Council of Medicine, and comprises a theoretical test, analysis of curriculum and an interview.

The residency program in Medical Genetics is available in only five Brazilian States (Minas Gerais, Bahia, São Paulo, Rio Grande do Sul, Rio de Janeiro) and in the Federal District. A total of 11 service providers offer 19 spots available for residents yearly. Based on registration in SBGM, there are currently less than 300 Medical Geneticists in the whole country.

### Genetic counseling

Genetic counseling is usually offered by medical geneticists and health professionals from different backgrounds, at genetic service providers usually associated with academia. The Biology Federal Council (“Conselho Federal de Biologia”) recognizes genetic counseling as a field of specialization, but SUS does not recognize these professionals or other nonmedical professionals as an integral part of a multidisciplinary healthcare team. With the recent guidelines established for integral attention given to rare disorders (Brazilian Ministry of Health, 2014; Box 1), after claims made by many professional categories and scientific societies, the national resolution will formally include nonmedical geneticists trained in Genetic Counseling in public health teams for providing genetic counseling. Accordingly, several courses in Genetic Counseling for health professionals are being formulated and will soon be implemented (Institute of Biosciences of the University of Sao Paulo as a professional postgraduate program - M. R. Passos-Bueno, pers. comm.; Brazilian Society of Medical Genetics, 2014). These programs will allow the training of a greater number of professionals, whom, together with the medical geneticists, will ensure better access to genetic services offered across the country.

## The Burden of Birth Defects and Genetic Disorders

The total birth prevalence of serious genetic congenital disorders in Brazil was estimated by the March of Dimes to be 57.2 per 1000 live births (Christianson et al. 2006). When classifying by mode of inheritance, estimates of defects per 1000 are as follows: 7 for dominant single gene, 1.3 for X-linked and 3.9 for recessive; 3.6 for chromosomal and 36.9 for congenital malformations in general (Christianson et al. 2006). The birth prevalence of neural tube defects has been estimated at 6390 new cases per year (Christianson et al. 2006).

No registry of birth defects or genetic disorders is available in Brazil. There are, however, some instruments that could provide some data. One of them is the field introduced in 2000 (CEInfo (Coordenação de Epidemiologia e Informação) 2011) for recording the presence of congenital malformation in the live-birth declaration (LD) – official document issued by hospitals. Nevertheless, the completion of this field is not mandatory, and consequently, underreporting of congenital anomalies has frequently been shown in several analyses (DATASUS; Cunha et al. 2002; Guerra et al. 2008a,b).

Exposure to teratogens during pregnancy is an important risk factor for these defects in Brazil. Studies have shown that misoprostol, a synthetic analog of prostaglandin E1 originally used to treat peptic ulcers but shown to increase the risk of Moebius syndrome and limb transversal defects if taken in pregnancy (Gonzalez et al. 1998; Dal Pizzol et al. 2006), still is widely used in Brazil to induce illegal abortion. As a result of the high prevalence of leprosy in Brazil, the use and prescription of thalidomide has been approved in Brazil since 1965, under a very restrictive regulation by the Federal Government (law no. 10.651, 2003). Nevertheless, births of individuals with thalidomide syndrome were reported in this century (Vianna et al. 2011). The use of alcohol in early pregnancy seems to accounts for a large proportion of birth defects, as well as intellectual disability in Brazil, as suggested by the estimated incidence of 38.7/1000 live births of fetal alcohol spectrum disorders in the city of São Paulo (Mesquita and Segre 2009). This scenario is partially related to the Brazilian culture of self-medication and the possibility of purchasing several prescription medications “over the counter”. A very positive initiative was the establishment of the National System of Information about Teratogen Agents (SIAT) in 1990 (Schüller-Faccini et al. 2001; Dal Pizzol et al. 2008) which was set up to better educate the population concerning the use of drugs and their possible teratogenic effects. It is, however, funded by a university and research grants.

The relevance of birth defects/genetic disorders in public health has become increasingly prominent since 2000, when the congenital malformations became the second cause of deaths in children younger than 1 year old. Congenital anomalies also became accountable for 20% of the deaths, exceeding the total sum between the 3rd and 4th causes, which are, respectively, related to respiratory and infectious diseases (DATASUS; Fig. 3). Birth defects/genetic disorders account for more than one-third of the total pediatric admissions in certain tertiary care hospitals in Brazil (Horovitz 2003; Horovitz et al. 2005). An increment of the permanency and hospitalization cost for this group of diseases can also be noticed (DATASUS, 2009). This information reinforces the importance of the implementation of the new policy for rare diseases in Brazil (Brazilian Ministry of Health, 2014; Box 1).

## Genetic Newborn Screening

Genetic population screening has been performed in Brazil only for newborns. It started with isolated initiatives in the 1970s and 1980s, without governmental directions or policies (Carvalho et al.^2007^; Leão and Aguiar 2008). In the 1990s, newborn screening became mandatory for some diseases, and SUS started to fund the tests needed for such procedures. Finally, in 2001, it was formally established by the Ministry of Health, became known as the National Newborn Screening Program (*Programa Nacional de Triagem Neonatal* – PNTN) and has been funded by the Federal government since then. Its specific goals are to ensure equitable access for all Brazilian newborns; organize State screening networks; ensure therapy and follow-up for each disorder detected; reduce morbidity and mortality; provide guidelines to standardize regional services provided; and create and support National Newborn Screening Database to collect epidemiological data. The following diseases are screened by the program: phenylketonuria (PKU), congenital hypothyroidism (CH), SCD and other hemoglobinopathies (SC), cystic fibrosis (CF), and, recently included, biotinidase deficiency (BD) and congenital adrenal hyperplasia (CAH) (Marques-de-Faria 2006; Diário Oficial da União 12 December 2012, 54–55). Such recommendation followed a rationale of progressive implementation phases (Phase I: PKU and CH, Phase II: PKU, CH and SC; Phase III: PKU, CH, SC and CF; Phase IV: BD and CAH), taking into account existing inequalities in health care structure. The referenced services for newborn screening are supposed to ensure not only the screening process, but also diagnostic confirmation and appropriate monitoring and treatment of screened patients. Currently, there are 15 States in Phase III and 12 States in Phase IV of the program (Brazilian Ministry of Health). PNTN becomes part of a social program named “Living without limit” – National Plan for the Rights of Persons with Disabilities, which integrates actions in various fields, focusing on people with disabilities. The proposal is that the PNTN include, in addition to the blood-tests, screening for neonatal deafness and ophthalmologic evaluation. By 2013, there were reference services registered by PNTN in all Brazilian States, providing coverage for 80% of newborns (Brazilian Ministry of Health, 2013). The program has shown good quality coverage, flexibility in the process, and also the possible integration capability between the primary health care and reference centers. The PNTN is run by municipal and state health offices; the global supervision is entrusted to the technical advisory group, established and coordinated by the Health Care Department of the Ministry of Health (Marques-de-Faria 2006; Brazilian Ministry of Health). A 10-year follow up study on the screening of hemoglobinopathies conducted in the state of Rio de Janeiro showed that early diagnosis and treatment of newborns resulted in substantial improvements in survival and quality of life of Brazilian children with SCD (Lobo et al. 2014). These results are quite encouraging and similar studies should be conducted so as to evaluate the effectiveness of the PTNT program in the health of these affected individuals across the different regions of the country.

The panel of diseases currently screened by the PTNT (Table 1) still is modest when compared with the screening programs of some developed countries. Nevertheless, PTNT represents the largest initiative carried out by SUS in the area of genetics (Leão and Aguiar 2008). Expanded neonatal screening can be alternatively performed by private laboratories, which offer screening through mass spectrometry for 30 rare treatable metabolic disorders at least.

Data on the prevalence of specific single gene disorders have become available thanks to the analysis conducted by the newborn screening program (Table 1). It has been shown that the incidence or prevalence of these diseases varies across the country (Cançado and Jesus 2007; Silveira et al. 2008; Stranieri and Takano 2009; Nunes et al. 2013). In some cases, they reflect the different proportions of European, African or Amerindian contribution to the population structure. For example, SCD was estimated to have a prevalence of 1:1000 live births by Cançado and Jesus (2007), but its prevalence was estimated as 1:650 in the state of Bahia in the Northeast, where African ancestry is the most preponderant, and of 1:13,000 in the Southern state of Rio Grande do Sul, with a high contribution of European ancestry (Cançado and Jesus 2007).

## Clinical Protocols in SUS

The Ministry of Health has issued several clinical protocols in an attempt at unifying the management of several disorders. In 2011, a special commission (CONITEC) was created with the aim to assist in the treatment as well as in the incorporation, exclusion or alteration of health technologies in the SUS. Today, 78 clinical protocols are available, excluding here the ones for cancer treatment. In this list, some genetic disorders are already contemplated (Table 1). It is of note that enzyme replacement therapy for Gaucher disease is contemplated and in 2011, 600 individuals were receiving the medication through the SUS. In turn, as the goals of SUS are to provide full, universal and free-of-charge medical access to the entire Brazilian population, the Government has been facing an increasing number of decisions from the Court of Law favoring the payment of expensive medications not considered as a regular medication by SUS.

## Pregnancy Termination and Assisted Reproduction

In Brazil, the legislation on this topic dates back to 1940 and it allows the physician to perform a pregnancy termination procedure only in cases in which the pregnancy imposes a life-threatening situation for the mother or if the pregnancy is the result of rape. If a woman terminates her pregnancy on her own or if the act is performed with her knowledge, she and the medical personnel responsible for the procedure could face 1–3 years in prison (according to Decreto-Lei no. 2848, 7 December 1940).

In April 2012, the Brazilian Supreme Court declared that terminating a pregnancy where the fetus is diagnosed with anencephaly is an anticipatory therapeutic act and could not be included in the established rules (“articles 124, 126 and 128, items I and II, of the Penal Code”) regulating pregnancy termination. Accordingly, in May 2012, the Federal Council of Medicine published recommendations for the physicians regarding the anticipatory therapeutic act in cases of unquestionable diagnosis of anencephaly. In these situations, the pregnant woman has the right to decide about the fate of her pregnancy without a Court authorization.

Assisted reproduction has been performed for several decades in Brazil, particularly in the private sector. However, there is no law regulating this procedure. In January 2011 (RESOLUTION CFM n° 2013/13), the Federal Medical Council implemented guidelines concerning this area. The technique is not supposed to select embryos based on the sex or other biological characteristic, except when used to avoid an X-linked disorder. The number of embryos implanted is related to the age of the mother: up to two embryos in mothers younger than 35 years old, three embryos, in mothers between 36 and 39 years old and four embryos in mothers between 40 and 50 years old. All the embryos not implanted in the uterus should be kept frozen and the parents should express their written will concerning the fate of these embryos in case of divorce, serious illness or death of one or both spouses or whether they wish to donate them. The reproductive technique can also be used for preventing and treating genetic or inherited disorders. In both cases, the couple must sign a written informed consent. Embryos kept frozen for more than 5 years of age could be discarded. In March 2005, a law (N° 11.105) issued by the National Bio Security Council (Conselho Nacional de Biossegurança – CTN-Bio) allowed the use of embryonic stem cells in research and therapy provided by human embryos produced by in vitro fertilization. For this purpose, the embryos must be regarded as nonviable or kept frozen for a minimum of 3 years and the parents must sign a written informed consent.

## Conclusions and Thoughts

The panorama of our 50 years of experience in human and medical genetics in Brazil shows a heterogeneous setting up of public and private genetic services across the country, with better infrastructure and development in the South and Southeast regions when compared to other regions of the country.

The regulations concerning rare diseases established by the Federal Government (Box 1) opened the possibility of conducting accredited clinical and genetic testing. It is now expected that network services will be established, which will increase the capacity of work of the current available trained professionals in genetics, who are still too few in number to attend to all of the Brazilian population.

The introduction of NGS will bring new perspectives in diagnosis and ethical issues. Its application will certainly reduce costs for establishing diagnosis, mainly of heterogeneous disorders, such as Noonan syndrome, Neuromuscular disorders, among several others, where the precise diagnosis depends on several laboratorial exams and analysis of several genes. Some Brazilian groups are currently implementing NGS facilities. As an example, the Human Genome Center and Stem Cell (HUGH-CEL), University of São Paulo, offers a NGS panel containing about 500 genes for 4 main groups of disorders (Neuromuscular/Neurodegenerative; Craniofacial/Skeletal dysplasias; treatable forms of IEM and Hereditary forms of Cancer). No guidelines have yet been formally established for conducting clinical exome or genome testing, as well as for informing incidental findings.

In summary, much has yet to be done to have public genetic services organized in Brazil. A balance between strengthening the genetic services already available and the creation of new ones, particularly in the neglected regions of Brazil, is a challenge that this new Federal Policy will face in the near future. Hopefully, planned and implementation actions should be taken by the Brazilian government together with specialists, such as scientists and geneticists affiliated with the two main Brazilian Genetic Societies (SBG and SBGM), and agents of rare/genetic disease patient/parent organizations. These actions will certainly have a significant impact on improving health care for the Brazilian population and research on medical genetics and genomics.

## Websites

Brazilian Ministry of Health – legislation: http://portalsaude.saude.gov.br/index.php/profissional-e-gestor/legislacao (n.d.). DATASUS (Department of Information and Informatics, Unified Health System,:Brazilian Ministry of Healthy): tabnet.datasus.gov.br (accessed 19 June 2014). IBGE – Instituto Brasileiro de Geografia e Estatística (Brazilian Institute of Geography and Statistics): http://www.ibge.gov.br (accessed 21 June 2014). PNTN—Programa Nacional de Triagem Neonatal (National Newborn Screening Program), report. 2007: http://portal.saude.gov.br/portal/arquivos/pdf/INDICADORES_ TRIAGEM_NEONATAL.pdf (n.d.). Brazilian Society of Newborn Screening: http://www.sbtn.org.br (accessed 23 June 2014). SIAT (Serviço de Informação Sobre Agentes Teratogênicos). 2011: http://gravidez-segura.org/ (n.d.). United Nations Brazil: http://hdr.undp.org/en/statistics/hdi (n.d.). World Bank: data.worldbank.org (n.d.).
